# Papillary Thyroid Carcinoma in Patients with Carotid Body Tumors: Prevalence and Management in a Retrospective Surgical Series from Two Tertiary Centers

**DOI:** 10.3390/jcm15051864

**Published:** 2026-02-28

**Authors:** Mohammed Alshahrani, Sharif Almatrafi, Alanoud Alshathri, Manar Alzahrani, Mohammed Alessa, Saleh Aldhahri, Majed Albarrak, Mohammed Almayouf, Khalid AlQahtani

**Affiliations:** 1 Department of Otolaryngology—Head and Neck Surgery, King Fahad Medical City, Riyadh 11525, Saudi Arabia; alanouda.alshathri@gmail.com (A.A.); majed.hns@gmail.com (M.A.); moalmayouf@kfmc.med.sa (M.A.); 2 Department of Otorhinolaryngology—Head and Neck Surgery, College of Medicine, Prince Sattam Bin Abdulaziz University, Alkharj 11942, Saudi Arabia; sharifalmatrafi@gmail.com; 3 Division of Otolaryngology—Head and Neck Surgery, Department of Surgery, King Abdulaziz Medical City, Ministry of National Guard Health Affairs, Riyadh 11481, Saudi Arabia; manaralkhatam@gmail.com; 4 Department of Otolaryngology—Head and Neck Surgery, College of Medicine, King Saud University, Riyadh 11461, Saudi Arabia; maalessa@ksu.edu.sa (M.A.); saldhahri@ksu.edu.sa (S.A.); kalqahtani@ksu.edu.sa (K.A.)

**Keywords:** carotid body tumor, papillary thyroid carcinoma, concurrent tumors, retrospective cohort study, thyroid nodules, tumor prevalence

## Abstract

**Background/Objectives:** The co-occurrence of a carotid body tumor (CBT) and papillary thyroid carcinoma (PTC) is a rare clinical event. The frequency of this dual pathology in recent reports has sparked a debate on whether it represents a true pathophysiological association or an artifact of increased diagnostic surveillance. This study aims to report the prevalence, clinicopathological characteristics, management, and outcomes of concurrent CBT and PTC in a contemporary cohort. **Methods:** We conducted a retrospective review of patients who underwent CBT resection at two tertiary centers between 2014 and 2024. Data on patient demographics, tumor characteristics, preoperative imaging, surgical management (single stage vs. staged), final histopathology, and clinical outcomes were collected and analyzed. **Results:** Overall, 32 patients with surgically resected CBTs were included. Eleven patients (34.4%) had thyroid nodules identified on preoperative imaging. The mean age of the participants was 57.2 ± 16.3 years. Females represented the majority of the population (*n* = 27, 84.4%). Nine patients underwent thyroid surgery with subsequent pathological confirmation. Management involved resection at two different time intervals in five cases (55.6%) and a single-stage operation in four (44.4%). On final pathology, PTC was confirmed in eight patients (25.0%). During the follow-up period, no recurrences of either tumor type were observed. **Conclusions:** The prevalence of concurrent PTC in patients with CBTs is significantly higher than previously reported, reaching 25% in our cohort. This incidental finding raises the possibility of surveillance bias or underlying genetic mechanisms. Management with either a single-stage or staged surgical approach was not associated with major complications. The prognosis for patients with this dual pathology is excellent and appears to be dictated by the independent characteristics of each tumor.

## 1. Introduction

Carotid body tumors (CBTs) are uncommon neck neoplasms that typically require detailed preoperative imaging for diagnosis and surgical planning [1]. As this imaging routinely encompasses the thyroid gland, incidental thyroid nodules are frequently detected during CBT workup [2]. Several reports have described the coexistence of CBTs and papillary thyroid carcinoma (PTC), raising uncertainty as to whether this reflects a true biological association or incidental detection related to intensified neck imaging [2,3,4].

Accounting for nearly 0.5% of all head and neck tumors, CBTs have a low annual incidence of approximately one case per 30,000 individuals. Therefore, the literature on CBTs is mostly derived from case reports and small series [5,6]. These tumors usually present as a painless neck mass that may be discovered incidentally during a routine clinical examination. High-resolution ultrasound plays a vital role in diagnosing CBTs, as it enables visualization of tumor characteristics, including hypoechoic masses with hypervascularity [7]. Furthermore, computed tomography (CT) angiography or magnetic resonance imaging (MRI) may be used to assess the extent of the tumor and its relationship with surrounding structures [8]. The complexity of the surrounding anatomical structures, mainly the carotid arteries and cranial nerves, carries substantial risks for surgical excision, which may dictate surgical options in bilateral cases [9,10].

Thyroid nodules are a common clinical finding with a high prevalence, ranging from 34% to 70%, using high-resolution ultrasound screening [11,12]. The majority of these nodules are benign (95%) [13]. For the minority that are cancerous, PTC constitutes the majority of thyroid cancers (85%) [14]. Various diagnostic methods are used for the diagnosis of thyroid nodules; fine needle aspiration (FNA) cytology is considered the gold standard for evaluating thyroid nodules [15,16]. High-resolution ultrasonography enables the characterization of the nodule, such as its size, composition (cystic vs. solid), and echogenicity [17,18]. In addition to reported cases of CBT and PTC, thyroglossal duct carcinoma, alongside CBT, has also been reported [19]. Such co-occurrences may be linked to shared genetic mutations, such as the RET proto-oncogene, which could contribute to the development of both tumor types [20,21]. Furthermore, the diagnostic workup for a neck mass often reveals both tumors through imaging and FNA owing to their anatomical proximity and similar clinical presentation, which leads to the discovery of one while investigating the other [22,23].

The extant literature is largely limited to case reports and small series, providing inconsistent estimates of prevalence and limited characterization of how concurrent thyroid findings are managed in patients undergoing surgery for CBTs [2,4]. In this study, we add to this growing body of evidence by describing the relatively high prevalence of imaging-detected thyroid nodules and pathology-confirmed PTC among a cohort of patients undergoing surgical resection for CBT at two tertiary centers. We further sought to characterize clinicopathological features and surgical management patterns in patients with concurrent thyroid disease identified during the diagnostic workup or perioperative evaluation of CBT. This study was not designed to establish causal associations but to provide a systematic descriptive account of this coexistence in a well-defined surgical cohort.

## 2. Materials and Methods

### 2.1. Study Design and Setting

This study was a retrospective cohort analysis conducted at two tertiary care centers in Riyadh, Saudi Arabia, and received institutional review board approval from King Fahad Medical City for both centers (IRB Log Number: 24-177). The requirement for individual patient consent was waived owing to the retrospective nature of the research. Data were collected locally at each center through review of electronic medical records. De-identified data were subsequently compiled for analysis, and no direct transfer of identifiable patient information occurred between institutions. All study procedures were conducted in accordance with institutional and national ethical standards for retrospective human research. Eligible patients were identified over a 10-year period, from January 2014 to January 2024. This report adheres to the STROBE guidelines for observational studies.

### 2.2. Participants

The source population included all patients with a histopathologically confirmed diagnosis of CBT. We included patients who underwent complete diagnostic investigation and subsequent surgical excision of their CBT at one of the participating centers. Patients were excluded if their primary treatment was performed at an outside institution, if they received non-surgical treatment such as radiation therapy, or if their medical records contained substantial missing data relevant to the primary study variables.

### 2.3. Diagnostic and Surveillance Pathway

Patients who presented with a neck mass underwent initial evaluation with contrast-enhanced CT of the neck to characterize the lesion. When a CBT was suspected, further evaluation included MRI angiography for vascular assessment. Thyroid nodules or masses identified incidentally on cross-sectional imaging prompted a dedicated high-resolution neck ultrasound, which was considered the primary modality for thyroid evaluation. Thyroid nodules were identified based on radiology reports. Nodules were risk-stratified using the American College of Radiology Thyroid Imaging Reporting and Data System (TI-RADS). When nodules met criteria for further evaluation, ultrasound-guided FNA was performed.

### 2.4. Variables and Data Sources

Data were extracted from the institutional electronic medical records. A standardized data collection sheet was used to record all relevant information. Two descriptive outcomes were evaluated in this study. First, the presence of a thyroid nodule was defined as any thyroid lesion detected on preoperative imaging performed as part of the CBT workup. Imaging modalities included high-resolution neck ultrasound and cross-sectional imaging (CT angiography or MRI), as documented in radiology reports. Second, PTC was defined as pathology-confirmed PTC among patients who underwent thyroid surgery, either at our institution or at an external center with complete histopathologic documentation available. Concurrent thyroid disease was defined as a thyroid nodule or malignancy identified during the diagnostic workup for CBT or within the same perioperative episode, rather than as a remote or unrelated diagnosis. Thyroid nodules were further categorized based on their FNA according to the Bethesda system of thyroid nodules. All identified nodules were managed according to the 2015 American Thyroid Association Management Guidelines for Adult Patients with Thyroid Nodules and Differentiated Thyroid Cancer. We defined ‘negative for malignancy’ as either the absence of nodules on imaging or the presence of radiologically benign, sub-centimeter nodules not meeting criteria for biopsy. TI-RADS scores were extracted from radiology reports, and when multiple nodules were present, the highest TI-RADS category per patient was recorded. Shamblin classification was determined based on preoperative radiologic assessment using CT and MRI findings, as documented in imaging reports. The main variables included baseline patient and tumor characteristics:

- Demographics: Age at diagnosis and sex.
- CBT characteristics: Shamblin classification (as determined by review of imaging and operative reports), tumor side, and final tumor size in centimeters on pathology.
- Clinical findings: Presence of palpable preoperative cervical lymph nodes.

For patients identified with thyroid nodules, we collected extra descriptive variables such as nodule size, preoperative FNA cytology results, TI-RADS score, final TNM staging (8th edition), PTC subtype, presence of lateral lymph node metastasis, laterality of the PTC relative to the CBT, and whether the surgical procedures for both tumors were performed in a single operation or were resected at two different time interval. Clinical outcomes, including tumor recurrence and the date of the last follow-up, were also recorded.

### 2.5. Follow-Up and Recurrence Assessment

Postoperative follow-up was conducted at regular intervals, typically twice per year during the first 3 years and annually thereafter. At each follow-up visit, patients underwent clinical evaluation and radiologic surveillance, including neck ultrasound and either contrast-enhanced CT or MRI. For patients with thyroid malignancy, laboratory testing was also performed as part of routine surveillance. Recurrence of CBT or thyroid carcinoma was defined as clinical or radiologic evidence of disease on follow-up evaluation. Patients without evidence of recurrence were censored at the date of last clinical or radiologic follow-up.

### 2.6. Statistical Analysis

Descriptive statistics were used to summarize the data. Continuous variables were presented as mean and standard deviation or median and interquartile range, while categorical variables were presented as frequencies and percentages. To compare baseline characteristics between patients with and without thyroid lesions, the two-sample t-test was used for continuous variables, and Fisher’s exact test was used for categorical variables. All analyses were conducted on a complete-case basis. A *p*-value < 0.05 was considered statistically significant. Statistical analysis was performed using R software (Version 4.3.3).

## 3. Results

### 3.1. Participant Characteristics

Thirty-two patients with a histopathologically confirmed diagnosis of CBT who underwent surgical excision were included in the final analysis; however, thyroid histopathology was only available for nine patients who had thyroid surgery. The mean age of the cohort was 57.2 ± 16.3 years, and the majority of patients were female (*n* = 27, 84.4%). On preoperative imaging, 11 patients (34.4%) were found to have thyroid nodules. The CBTs were classified according to the Shamblin system as Class I (*n* = 15, 46.9%), Class II (*n* = 12, 37.5%), and Class III (*n* = 5, 15.6%). Review of medical records showed no consistent comorbidities linked to chronic hypoxia (e.g., chronic lung disease, cyanotic congenital heart disease, or high-altitude residence). Obstructive sleep apnea was not systematically recorded. No patients had documented hereditary endocrine syndromes (e.g., MEN2, Cowden, hereditary paraganglioma) or prior diagnoses of paraganglioma or pheochromocytoma before CBT presentation.

### 3.2. Prevalence and Clinicopathological Findings of Thyroid Lesions

All patients underwent cross-sectional neck imaging as part of CBT evaluation. Initial assessment most commonly included contrast-enhanced CT of the neck, followed by MRI angiography for vascular characterization. Thyroid nodules or masses were identified incidentally during review of these imaging studies, prompting a dedicated high-resolution neck ultrasound for further thyroid evaluation. Of the 11 patients identified with thyroid nodules on imaging, nine underwent thyroid surgery. Eight patients were evaluated and surgically managed at our institution, while one patient underwent thyroid surgery at an external center and was subsequently referred to our facility with comprehensive medical documentation for CBT surgery. Two patients with thyroid nodules exhibited no high-risk features on ultrasound imaging; therefore, subsequent investigations were not carried out. Final pathology confirmed PTC in eight of these patients and a benign follicular nodule in one, yielding an overall PTC prevalence of 25.0% (8 of 32) in the entire CBT cohort. Preoperative FNA was performed on eight of the nodules. The results were diagnostic of or suspicious for PTC (Bethesda V/VI) in five cases (62.5%), benign (Bethesda II) in two cases (25.0%), and showed follicular lesion of undetermined significance (Bethesda III) in one case (12.5%). Among the patients who underwent thyroid surgery, 88.9% (8 of 9) were confirmed to have PTC on final pathology.

### 3.3. Comparison of Baseline Characteristics

Baseline characteristics were compared between patients with and without thyroid nodules on imaging (Table 1). No statistically significant differences were observed between the two groups with respect to age, sex, Shamblin classification, CBT side, or CBT size.

### 3.4. Management and Final Pathology of Thyroid Lesions

The clinicopathological details of the nine patients who underwent thyroid surgery are summarized in Table 2. The mean nodule size was 2.9 ± 1.8 cm. Surgical resection of the CBT and the thyroid lesion was performed at two different time intervals in five patients (55.6%) and as a single-stage operation in four patients (44.4%). For the eight patients diagnosed with PTC, five (62.5%) presented with T1N0 stage disease. The classic PTC subtype accounted for seven cases (87.5%). Pathological evidence of lateral neck lymph node metastasis was present in two patients (25.0%). The thyroid cancer was located contralateral to the CBT in four cases (57.1%) and on the same side in three cases (42.9%). No reports of major complications were found, such as massive bleeding, blood transfusions, stroke, or cranial nerve weakness. All nine patients completed the described postoperative surveillance protocol, with a median follow-up duration of 50 months (range: 9–120 months). During this period, no recurrences of either carotid body tumor or papillary thyroid carcinoma were observed.

## 4. Discussion

In this cohort of 32 patients with surgically resected CBTs, 11 (34.4%) were found to have thyroid nodules on preoperative imaging. Of these, nine underwent thyroid surgery, with final pathology confirming PTC in eight patients. This resulted in an overall PTC prevalence of 25.0% for the entire cohort and a 88.9% rate of PTC among patients with identified nodules. A comparison between patients with and without thyroid nodules on imaging revealed no statistically significant differences in baseline characteristics, including age (mean 62.3 vs. 54.9 years) or sex. Management of the nine patients with confirmed thyroid nodules was individualized, with five (55.6%) undergoing resection of both tumors at two different time intervals and four (44.4%) having a single-stage operation. No recurrences of either CBT or PTC were observed during the follow-up period.

The prevalence of concurrent thyroid cancer alongside CBTs in our cohort is discordant with the prevailing literature, which often characterizes this co-occurrence as extremely rare [3], with reported rates as low as 3.7% in larger series [23]. The most immediate explanation is pervasive surveillance bias, where the diagnostic workup for a CBT necessitates meticulous neck imaging, including high-resolution ultrasound and CT angiography. This inevitably turns a focused investigation into a broad screening exercise, capturing incidentalomas. This is supported by the fact that carotid Doppler is a leading modality for the incidental discovery of thyroid cancer [24], and cases of PTC directly mimicking CBTs on angiography have been documented [22,23]. However, attributing our finding solely to detection bias may be an oversimplification. The exclusive occurrence of thyroid lesions in female patients in our cohort, alongside trends linking them to higher Shamblin classes, might hint at underlying biological or demographic factors. This raises the possibility of a shared, yet elusive, genetic predisposition or a common environmental trigger in certain populations [4,23].

For context, prior studies of incidental thyroid nodules detected on cross-sectional imaging in non-thyroid populations report malignancy rates of approximately 3–4.9%. Although our observed proportion appears higher than these background estimates, the absence of an internal control group precludes formal comparison [25]. Accordingly, the present findings should be interpreted as descriptive rather than evidence of an increased risk. This interplay between incidental discovery and potential true association directly influences the diagnostic paradigm for neck masses and the use of FNA. For thyroid nodules, ultrasound-guided FNA remains the unequivocal gold standard, providing a reliable, preoperative diagnosis of PTC [4,23]. In contrast, FNA is strongly contraindicated for a suspected CBT owing to the significant risks of hemorrhage in these hypervascular tumors, potential catastrophic injury to the carotid vessels, and the possibility of provoking a hypertensive crisis in hormonally active lesions [1,22,26]. Consequently, the diagnostic workup for a CBT relies exclusively on advanced cross-sectional imaging (CT/MRI angiography) to define the anatomy and vascularity, with histopathological confirmation reserved for the surgical specimen [22,27]. This creates a clinical scenario where the same imaging study ordered to characterize a CBT, such as a high-resolution neck ultrasound or CT angiography, simultaneously serves as a screening tool for thyroid nodules. This may lead to the discovery of incidental thyroid findings, precisely as observed in our cohort. It should be noted that the present study did not quantify imaging frequency, intensity, or modality-specific yield; the previous section should be interpreted as hypothesis-generating.

While surveillance bias may offer one explanation for our observed prevalence, the possibility of a true association remains an intriguing perplexity. Po et al. (2020) [23] emphasize that, in regions where the incidences of CBTs and thyroid cancer are independently high, the probability of their co-occurrence within the same individual remains exceedingly low unless a true syndromic relationship exists. This framework underscores that even in the context of regional clustering, simultaneous presentation is unlikely without a unifying pathogenic mechanism [4]. Chronic hypoxia, the only well-established acquired risk factor for CBTs and paragangliomas, is a major driver in this model [4,26]. It directly promotes tumorigenesis in the hypoxia-sensitive chemoreceptor cells of the carotid body by dysregulating hypoxia-induced genes and pathways [4]. While a direct hypoxic mechanism in the pathogenesis of PTC has not been clearly defined, the reported coexistence of PTC and CBTs raises the question of a potential underlying genetic or hereditary association. However, a definitive genetic link has not yet been established [4].

The search for a unified genetic explanation remains complex. The well-defined syndromic link between paragangliomas and medullary thyroid carcinoma via germline RET point mutations in MEN2 syndromes is not translatable to PTC. PTC originates from follicular cells and is more commonly associated with somatic BRAF mutations or RET/PTC rearrangements [4,28]. Current evidence suggests no single, common germline mutation directly predisposes to both CBT and PTC. Instead, the most promising link involves the succinate dehydrogenase (SDH) gene complex. Germline mutations in SDHx genes are central to the etiology of hereditary paragangliomas [3,29] and have also been associated with a broader tumor predisposition, including PTC, in Cowden syndrome-like phenotypes [3,30,31]. This suggests that an inherited SDHx mutation could be the first hit, predisposing a patient to both tumors, with individual somatic events (second hits) like loss of heterozygosity in the carotid body or somatic SDHC/D loss or BRAF mutation in the thyroid, driving independent tumorigenesis in each tissue [3,32]. Therefore, the co-occurrence in our cohort may represent a shared but indirect genetic predisposition mediated by genes such as SDHB that create a permissive environment for multiple cancer types.

All cases with concurrent CBTs and thyroid nodules were females in our study. Epidemiological data indicate a significant female predominance in the incidence of CBTs, with women comprising a substantial majority of cases during the sixth and seventh decades of life [33]. This gender disparity extends to the development of additional primary neoplasms; research has noted that among patients with CBTs who developed second primary tumors, thyroid cancer occurred exclusively in females [33]. Furthermore, primary thyroid paragangliomas, often misdiagnosed as thyroid malignancies, exhibit an even more drastic female-to-male ratio of approximately 8:1 [34,35]. These findings suggest that female sex may be an intersectional factor, associated not only with a higher prevalence of CBTs but also with a specific susceptibility to concurrent thyroid carcinomas or paragangliomas mimicking malignancy [36].

Management of patients with concurrent CBT and PTC necessitates an individualized approach, where surgical strategy was nearly evenly split between single-stage (44.4%) and staged (55.6%) procedures. This methodology is supported by literature demonstrating that a simultaneous resection is both feasible and safe in carefully selected cases, characterized by younger patient age, favorable tumor biology (e.g., lower Shamblin class CBTs and smaller, low-risk PTCs), and a comprehensive preoperative diagnostic workup that allows for meticulous planning [4,23]. The decision to stage procedures, which was slightly more prevalent in our study, is primarily based on the patient’s medical condition and preference. By separating the procedures, surgeons can avoid significant blood loss, nerve injury, and prolonged operative time, thereby reducing overall patient morbidity, particularly in older individuals or those with more technically challenging tumors [37].

Surgical outcomes for thyroid nodules classified as Bethesda category III (AUS/FLUS) and category II often reveal malignancy rates exceeding original Bethesda System estimates. For Bethesda category III nodules, institutional and registry data indicate malignancy rates ranging from approximately 26% to nearly 43% in surgically resected cases, significantly higher than the initially predicted 5–15%, with Papillary Thyroid Carcinoma representing the predominant histologic subtype [38,39,40]. Malignancy risk within this category is elevated in certain demographics, such as men aged 31 to 45 who may exhibit rates exceeding 50%, and is further predicted by independent risk factors including microcalcifications, irregular shape, smaller nodule diameter (<1 cm), and specific cytological subcategorization where atypia of undetermined significance (AUS) carries a higher risk than follicular lesion of undetermined significance (FLUS) [39,40,41]. Likewise, while Bethesda category II nodules are managed conservatively, patients selected for surgery demonstrate a malignancy rate of 12.7%, dropping to 7.8% when incidental microcarcinomas are excluded, which suggests that clinical selection bias may identify a higher-risk cohort than the general population [41].

The excellent oncologic outcomes in our cohort, with no recurrences of either CBT or PTC during follow-up, align with reports of low recurrence rates following complete resection; however, this excludes malignant or fibrous variants [23,42]. The absence of PTC recurrence is consistent with the specific findings of Po et al. [23] in a similar patient group and is statistically plausible given that the majority of our PTC cases (62.5%) presented with early-stage (T1N0), classic subtype disease, which carries a very favorable prognosis, where recurrence is a rare event [43]. While our limited sample size and follow-up period preclude definitive conclusions, these outcomes suggest that both single-stage and staged surgical strategies, when appropriately selected, can achieve definitive control for this patient population. Moreover, our study is subject to verification bias inherent to retrospective surgical series. Pathologic confirmation was restricted to patients who underwent thyroidectomy, representing a non-random subset selected based on suspicious sonographic features or indeterminate cytology. No germline or somatic genetic testing was performed; therefore, any genetic mechanism remains speculative and cannot be evaluated in the present study. Finally, inclusion of a patient whose thyroid surgery was performed at an external institution may introduce heterogeneity in diagnostic workup and management. Future multi-institutional studies with larger cohorts and extended follow-up are warranted to validate the underlying mechanisms, long-term outcomes, and to refine the optimal selection criteria for single-stage versus staged resection.

## 5. Conclusions

The high prevalence of PTC observed in our cohort of patients with CBT is a significant finding that challenges its characterization as an exceedingly rare phenomenon. This may be attributable to a combination of intense surveillance bias, where advanced neck imaging for CBT workup incidentally detects thyroid nodules and might be a true association linked to shared environmental factors such as chronic hypoxia or a predisposing genetic background, including SDHx mutations. The excellent oncological outcomes achieved, with no recurrences in our cohort, demonstrate that both single-stage and staged procedures are viable strategies when carefully selected.

## Figures and Tables

**Table 1:** Baseline Clinicopathological Characteristics of Patients with Carotid Body Tumors, Stratified by the Presence of Thyroid Nodules on Imaging.

Characteristic	Thyroid Nodules Absent (*n* = 21)	Thyroid Nodules Present (*n* = 11)	*p*-Value
**Age, years**	54.9 ± 17.7	62.3 ± 12.0	0.227 ^1^
**Gender**			0.138 ^2^
Male	5 (23.8)	0 (0.0)	
Female	16 (76.2)	11 (100.0)	
**Shamblin Classification**			0.287 ^2^
Class I	12 (57.1)	3 (27.3)	
Class II	6 (28.6)	6 (54.5)	
Class III	3 (14.3)	2 (18.2)	
**Preoperative Lymph Nodes**			0.111 ^2^
No	21 (100.0)	9 (81.8)	
Yes	0 (0.0)	2 (18.2)	
**Side of Carotid Body Tumor**			0.139 ^2^
Right	10 (47.6)	2 (18.2)	
Left	11 (52.4)	9 (81.8)	
**Size of CBT on Pathology, cm**	3.9 ± 2.0	3.7 ± 1.8	0.817 ^1^

Data are presented as *n* (%) or mean ± standard deviation. Percentages for categorical variables are calculated as column percentages. ^1^ *p*-value calculated using the Two-Sample *t*-test. ^2^ *p*-value calculated using Fisher’s exact test.

**Table 2:** Clinicopathological and Management Characteristics of Patients with Thyroid Nodules on Final Pathology (*n* = 9).

Characteristic	Value
**Nodule Size, cm** ^1^	
Mean (SD)	2.9 (1.8)
Median [IQR]	2.4 [1.7–3.7]
**Preoperative FNA Diagnosis** ^1^	
Suspicious for/PTC	5 (62.5%)
Benign Follicular	2 (25.0%)
FLUS	1 (12.5%)
**TIRADS Score** ^1^	
TIRADS 4	7 (87.5%)
TIRADS 3	1 (12.5%)
**Surgical Approach**	
Staged Procedure	5 (55.6%)
Single-Stage Procedure	4 (44.4%)
**Details of PTC Cases (*n* = 8)**	
**TNM Stage** ^1^	
T1N0	5 (62.5%)
T3N0	1 (12.5%)
T3N1b	1 (12.5%)
T4N1b	1 (12.5%)
**PTC Subtype** ^1^	
Classic	7 (87.5%)
Other	1 (12.5%)
**Lateral Lymph Node Metastasis** ^1^	
Absent	6 (75.0%)
Present	2 (25.0%)
**Side of Thyroid Cancer** ^2^	
Left	3 (42.9%)
Right	2 (28.6%)
Isthmus	2 (28.6%)
**Tumor Laterality vs. CBT** ^2^	
Contralateral	4 (57.1%)
Same Side	3 (42.9%)
**Bethesda Score**	
Bethesda II	2 (25.0%)
Bethesda III	1 (12.5%)
Bethesda V/VI	5 (62.5%)

Data are presented as *n* (%) or mean (SD) and median [IQR]. Abbreviations: CBT, Carotid Body Tumor; FNA, Fine-Needle Aspiration; FLUS, Follicular Lesion of Undetermined Significance; IQR, Interquartile Range; PTC, Papillary Thyroid Carcinoma; SD, Standard Deviation; TIRADS, Thyroid Imaging Reporting and Data System; TNM, Tumor, Node, Metastasis. ^1^ Based on 8 patients with available data. ^2^ Based on 7 patients with available data.

## Data Availability

All data are available upon reasonable request.

## Abbreviations

CBT	Carotid body tumor
PTC	Papillary thyroid carcinoma
FNA	Fine-needle aspiration
CT	Computed tomography
MRI	Magnetic resonance imaging
TI-RADS	Thyroid imaging reporting and data system
TNM	Tumor–node–metastasis
SDH	Succinate dehydrogenase
SDHx	Succinate dehydrogenase gene complex
OR	Odds ratio
CI	Confidence interval
MEN2	Multiple endocrine neoplasia type 2
